# Heterogeneous Association of Chinese Adolescents’ Engaged Living With Problematic Internet Use: A Mixture Regression Analysis

**DOI:** 10.3389/fpsyg.2020.526290

**Published:** 2021-01-20

**Authors:** Jieting Zhang, Can Jiao, Chengfu Yu, Tianqi Qiao, Zhirong Li

**Affiliations:** ^1^College of Psychology, Shenzhen University, Shenzhen, China; ^2^College of Psychology, Guangzhou University, Guangzhou, China

**Keywords:** mixture regression analysis, problematic internet use, engaged living, social integration, absorption

## Abstract

The present study explored heterogeneity in the association between engaged living (i.e., social integration and absorption) and problematic Internet use (PIU). This study included 641 adolescents from four junior-senior high schools of Guangzhou, China. Besides the standard linear regression analysis, mixture regression analysis was conducted to detect certain subgroups of adolescents, based on their divergent association between engaged living and PIU. Sex, age, and psychological need were further compared among the latent subgroups. The results showed that a mixture regression model could account for more variance of PIU than a traditional linear regression model, and identified three subgroups based on their class-specific regression of PIU to engaged living. For the *High-PIU* class, lower social integration and higher absorption were associated with increased PIU; for the *Medium-PIU* class, only high social integration was linked with the increase of PIU. For the *Low-PIU* class, no relation between engaged living and PIU were found. Additionally, being male or having a lower level of satisfied psychological needs increased the link between engaged living and PIU. The results indicated a heterogeneous relationship between engaged living and PIU among adolescents, and prevention or intervention programs should be tailored specifically to subgroups with moderate or high levels of PIU and to those with lower levels of psychological needs’ satisfaction, as identified by the mixture regression model.

## Introduction

Problematic Internet use (PIU), an online behavior that has a negative effect on psychological, social, school, and/or work situations (Beard and Wolf, 2001), has become a serious problem for a large proportion of Chinese adolescents (Ni et al., 2017). Some empirical research has indicated that PIU is related to various negative developmental results among children and adolescents, such as subjective insomnia or poor sleep quality (Guo et al., 2018), and even psychological and physical symptoms, such as depression, emotional problems, attention-deficit hyperactivity disorder (Leménager et al., 2018), and suicidal behavior (Guo et al., 2018). Additionally, PIU was negatively associated with life satisfaction (Sha et al., 2019), such as satisfaction with the self, social relationship with families and friends (Tateno et al., 2019), and satisfaction with the living environment (Masten, 2001). Therefore, identifying the possible risk factors of PIU is of great importance for future prevention and intervention programs.

Engaged living, proposed by Froh et al. (2010) is defined as being passionate about helping others and being connected with others (social integration), and being completely immersed in activities (absorption). Masten (2001) suggested that living an engaged life would be beneficial in helping youths avoid poor choices that result in delinquent behaviors and thus increase their resilience to addictive behavior. Specifically, social integration, as an ability of engaging in social networks and life, has a positive effect on social relations, which is suggested to be a key factor of PIU (Prievara et al., 2018). PIU is positively related to social relational styles such as low social skill (Chi-Ying, 2018) and shyness (Prievara et al., 2018). People with high sociality and shyness prefer computer-mediated communication as a less anxious way of social interaction, which would induce higher PIU (Bolat et al., 2018). Social support gained from social integration would also protect adolescents from PIU, through increasing their motivation to engage in a healthy lifestyle, as well as reducing boredom and loneliness (Mo et al., 2018).

Absorption, as a positive predictor of emotional and psychological well-being (Philippe et al., 2009; Froh et al., 2010; Rivkin et al., 2018), would be a protective factor against PIU. People tend to experience flow and positive emotions when highly absorbed in activities with higher general positive affect and lower negative affect (Hernández et al., 2019), and fewer relational conflicts (Séguin-Lévesque et al., 2003). People who are absorbed in activities have positive social relationships with harmonious passions, and possibly have lower levels of PIU. However, there are some contrasting findings that flow experience, as generated by absorption, is a risk factor of PIU (Stavropoulos et al., 2018; Barnes et al., 2019). When people are in a state of flow, they feel intrinsically cheerful and self-reinforcing, but this is also accompanied with reduced self-consciousness, which may induce excessive use of the internet (Stavropoulos et al., 2018).

Problematic internet use and its associated factors are found to differ by gender (Baloğlu et al., 2018; Hu et al., 2019; Tateno et al., 2019) and age (Stavropoulos et al., 2018). Moreover, the link between PIU and engaged living would be associated with the degree to which one’s needs are being satisfied. From the perspective of self-determination theory (Davids et al., 2016), being pathologically involved with the Internet is involved with pursuing the needs for belonging, relationships, self-actualization, achievement, and mastery. Need satisfaction in daily real life predicted lower Internet use (Ataşalar and Michou, 2019). Self-esteem, which is related to considering oneself competent, has been found to mediate the link between social relationships and PIU (Park et al., 2014). In addition, self-control, as related to autonomous motivation, has been found to moderate the link between social relationships and PIU (Li et al., 2013). Taken together, besides demographic factors (e.g., gender and age), how engaged living influences PIU may be related to whether an adolescent’s psychological needs have been satisfied or not.

Methodologically, the standard linear regression model assumes the association between engaged living and PIU is homogenous, that is, it assumes the same regression model holds for all individuals. In reality, there may be considerable variability where some factors are important for certain subgroups of individual but not for others. Mixture regression models provide for a set of latent subgroups (i.e., classes) in which parameters of the regression model differ. This analytic approach provides a way to account for heterogeneity in association between predictors and outcome (Lanza et al., 2014).

Hence, the aim of the present study was to examine heterogeneity among Chinese adolescents in the association of PIU with engaged living, including social integration and absorption, and to investigate the relation of these associations with age, sex, and psychological need. We hypothesized that a few subgroups would be identified with different associations between engaged living and PIU; age, sex, and psychological need would differ across the subgroups.

## Materials and Methods

### Participants

Data were collected from four junior-senior high schools of Guangzhou, China. Guangzhou possesses one of the most advanced Internet infrastructures in the Chinese Mainland, and Internet use is pervasive in adolescents’ home and school lives. The study was approved by the Human Research Ethics Committee of each of the four schools. Seven hundred students in the four schools were randomly contacted with an invitation letter and information about the sampling procedures. Statistics collection was carried out by trained postgraduate students after obtaining written consent from the schools and parents and oral agreement from the participants with prior approval. All participants were required to complete the questionnaires under the unified guide, which stressed the need for authenticity, independence, and continuity of all answers. Participants were also told that they could choose not to answer any questions in the questionnaires and that they were free to withdraw from the study at any time during data collection. Participants had about 20 min to finish the questionnaires, and they received pencils as a reward after finishing the questionnaires. Fifty-nine participants were excluded for not completing the questionnaire carefully. The valid sample consisted of 641 adolescents (61.8% female, 74% of participants’ parents had only received senior school or lower education) from the 7th to 11th grades (*M*_age_ = 15.62 years, *SD* = 1.82).

### Measurements

#### Problematic Internet Use

The main dependent variable of the current study was PIU, measured by the Chinese version of the PIU Questionnaire (Li et al., 2010), the items of which were selected from Young’s (1998) Internet Dependency Questionnaire. Participants were asked to complete the 6-point Likert scale ranging from 1 (*not at all true*) to 6 (*always true*) with 10 questions based on their real conditions. For each of the 10 items, adolescents expressed their true thinking about each statement about themselves on a 6-point Likert-type scale ranging from 1 (*not at all true*) to 6 (*always true*). Mean scale score was computed across all 10 items; higher scores indicate higher PIU level. This measure has demonstrated good reliability and validity in Chinese adolescents (Li et al., 2013; Zhou et al., 2017). Young’s questionnaire has good construct validity (Kelley and Gruber, 2010) and predictive validity over internet addiction disorder (Zhang et al., 2019). Construct validity for the current data was tested by comparing five alternative models (Laconi et al., 2019). The confirmatory factor model indicated one-factor model had a moderate model fit (*x*^2^ = 236.02, *df* = 35, *x*^2^*/df* = 6.74, RMSEA = 0.095, CFI = 0.91, TLI = 0.88), and bifactor model with two specific dimensions had a slightly better fit (*x*^2^ = 148.93, *df* = 28, *x*^2^*/df* = 5.32, RMSEA = 0.08, CFI = 0.94, TLI = 0.91), which is similar to the previous literature. Descriptive analysis and reliability for all the measurements were shown in Table 1. Convergent validity was poor for this questionnaire, as indicated by the average variance extracted (AVE) below 0.5 (Fornell and Larcker, 1981), but reliability was fairly good for this scale.

**Table 1:** Descriptive statistics for the investigated variables.

	# of Item	Reliability			Average varianceextracted	*Mean*	*SD*
		Cronbach’s alpha	Compound reliability	MacDonalds omega			
Problematic internet use	10	0.86	0.86	0.88	0.39	2.27	0.86
Social integration	9	0.80	0.81	0.85	0.32	4.71	0.70
Absorption	6	0.78	0.79	0.84	0.40	4.76	0.75
Competence	6	0.55	0.52	0.67	0.16	4.16	0.80
Autonomy	7	0.70	0.61	0.69	0.20	4.28	0.86
Relatedness	8	0.75	0.76	0.81	0.29	5.30	0.88

#### Engaged Living

Engaged living, considered as a predictor of PIU, was assessed using the Engaged Living in Youth Scale (Froh et al., 2010). The ELYS is a 15-item measure of positive psychological functioning using a Likert type scale ranging from 1 (*definitely not like me*) to 6 (*exactly like me*). The ELYS assesses social integration (e.g., “I feel like a part of my community/neighborhood”) and absorption [e.g., “While doing my hobbies (e.g., sports, reading, musical instruments, acting), I feel ‘in the zone’.”]. An average score for all the items of each subscale were created. This measure has demonstrated good construct validity and good predictive validity over life satisfaction and prosocial behavior (Froh et al., 2010). The confirmatory factor model provided a moderate fit for the current data (*x*^2^ = 634.8, *df* = 89, *x*^2^*/df* = 7.13, RMSEA = 0.098, CFI = 0.82, TLI = 0.78). Although reliability was moderate for the two subscales, convergent validity was poor, as indicated by AVEs below 0.5; discriminant validity was poor for social integration and fair for absorption, as indicated by the comparison with the squared correlation coefficient between the two subscales (0.40; Fornell and Larcker, 1981).

#### Individual Characteristics

Age, sex, and psychological need were included to describe the characteristics of individuals in each latent class. Psychological need was assessed by the Basic Psychological Needs Scale (Deci and Ryan, 2000), which includes three factors: Competence (six items), which involves feeling a sense of mastery in one’s activities; autonomy (seven items), which is feeling that one’s choices and activities are self-determined as opposed to being controlled by internal or external pressures; and relatedness (eight items), which involves feeling that satisfying and meaningful connections are being made with others. Responses are provided for the 21 items (e.g., “I think I can freely determine my lifestyle”) using a 7-point Likert scale ranging from 1 (*not at all true*) to 7 (*very true*), and an average score for all the items were created. This measure has demonstrated good construct validity (Liu et al., 2013) and predictive validity over school well-being (Tian et al., 2014). The confirmatory factor model provided a marginal fit for the current data (*x*^2^ = 1200.2, *df* = 186, *x*^2^/*df* = 6.45, RMSEA = 0.092, CFI = 0.66, TLI = 0.61). The AVEs of the three subscales were below 0.5, and generally less than the squared correlation coefficients of a specific subscale with other subscales (0.30 and 0.18 for competence, 0.30 and 0.31 for autonomy, and 0.31 and 0.18 for relatedness), indicating poor convergent and discriminant validity.

### Analytic Strategy

First, tradition linear regression was used to examine the overall association between engaged living (including absorption and social integration) and PIU. Second, mixture regression analysis was used to identify subgroups of individuals with different patterns of association between predictors and outcome. Third, chi-square test and ANOVA were used to examine the association of class membership with age, sex, and psychological need. SPSS 20.0 was used for linear regression, chi-square test, and ANOVA, and LatentGold 4.5 was used for mixture regression analysis.

Specifically, mixture regression analysis accounts for heterogeneity in the overall regression coefficients by building a model with class-specific regression coefficients for each subgroup, without a known priori. To select the optimal mixture regression model, the Akaike information criteria (AIC) and Bayesian information criteria (BIC) were used; smaller AIC and BIC values indicate better balance between fit and parsimony (Zhang et al., 2014). Maximum-likelihood estimate was used for all mixture regression models, and significance tests for regression coefficients were conducted with Wald statistics.

## Results

### Standard Regression Analysis

To understand the overall association between engaged living and PIU, linear regression was used to relate PIU to social integration and absorption. Social integration was negatively related to PIU (*B* = −0.213, *t* = −3.455, *p* < 0.01) and absorption was positively related to PIU (*B* = 0.129, *t* = 2.242, *p* < 0.05). However, predictors only accounted for 1.8% of the total variance in PIU. Mixture regression was further applied to investigate the heterogeneity of the association within different classes of the participants.

### Mixture Regression Analysis

To determine the optimal number of classes, a mixture regression model with one to six classes was considered. As shown in Table 2, the lowest AIC suggested a 5-class model, while the lowest BIC suggested a 3-class model. One of the classes in the 5-class model accounted for only 2.17% of the sample, which had low statistical power for the identified class. AIC has been found to overestimate class number (Zhang et al., 2014). Taken together, a 3-class model was chosen as the optimal model. The percentages of variance explained for Classes 1, 2, and 3 were 6.3, 1.99, and 3.3%, respectively. The percentage of variance explained in each latent class was generally higher than the total explained variance in the standard regression model. This suggests there is considerable heterogeneity in the association of social integration and absorption with PIU.

**Table 2:** Comparison of competing mixture regression models.

	LL^a^	BIC^b^	AIC^c^
1-Class Regression	−805.18	1636.22	1618.37
2-Class Regression	−775.73	1609.62	1569.46
3-Class Regression	−756.55	1603.58	1541.10
4-Class Regression	−742.58	1607.96	1523.17
5-Class Regression	−734.53	1624.17	1517.06
6-Class Regression	−733.74	1654.91	1525.48

a LL: Log Likelihood.

b BIC: Bayesian information criteria.

c AIC: Akaike information criteria.

Regression coefficients for each class are shown in Table 3. Generally, the three classes differed significantly from each other in their intercepts (*Wald* = 29.97, *p* < 0.001) and regression coefficient of social integration (*Wald* = 9.30, *p* < 0.01), and marginally in the regression coefficient of absorption (*Wald* = 5.45, *p* = 0.066). Specifically, Class 1 (*B* = 2.97) and 2 (*B* = 3.03) had a much higher intercept of PIU than Class 3 (*B* = 1.37). Class 1 (*B* = −0.35) and 2 (*B* = −0.22) had a stronger negative effect of social integration than Class 3 (*B* = −0.04). Class 1 (*B* = 0.33) had a stronger positive effect of absorption than Class 2 (*B* = 0.22) and 3 (*B* = 0.22). Class 1, as the largest subgroup (42.6%), was defined by a negative association of social integration and a positive association of absorption with PIU. Class 2 (38.9%) was defined by a negative association of social integration while there was no significant association of absorption with PIU. Class 3 (18.5%) was defined by no association of social integration or absorption with PIU. Notably, there was significant difference (*F* = 1024.41, *p* < 0.001; *ps* < 0.001 for *post hoc* tests) in the level of PIU among Class 1 (*M* = 3.2, 95% CI [3.12, 3.28]), Class 2 (*M* = 2.04, 95% CI [2.01, 2.08]), and Class 3 (*M* = 1.25, 95% CI [1.22, 1.28]). Therefore, the three classes were subsequently labeled as the *High*-, *Medium*-, and *Low-PIU* classes.

**Table 3:** Parameter estimates for mixture regression model.

	Class 1 (42.6%): *High-PIU*		Class 2 (38.9%): *Medium-PIU*		Class 3 (18.5%): *Low-PIU*		Wald (=)
	*B*	z-value	*B*	z-value	*B*	z-value	
Intercept	2.97	6.42	3.03	9.57	1.37	6.71	29.97^***^
Social integration	−0.35^**^	−2.60	−0.22^**^	−2.69	−0.04	−0.97	9.30^**^
Absorption	0.33^**^	2.69	0.02	0.20	0.02	0.48	5.45

^*^p < 0.05, ^*^p < 0.01, ^***^p < 0.001.

**Figure 1:**
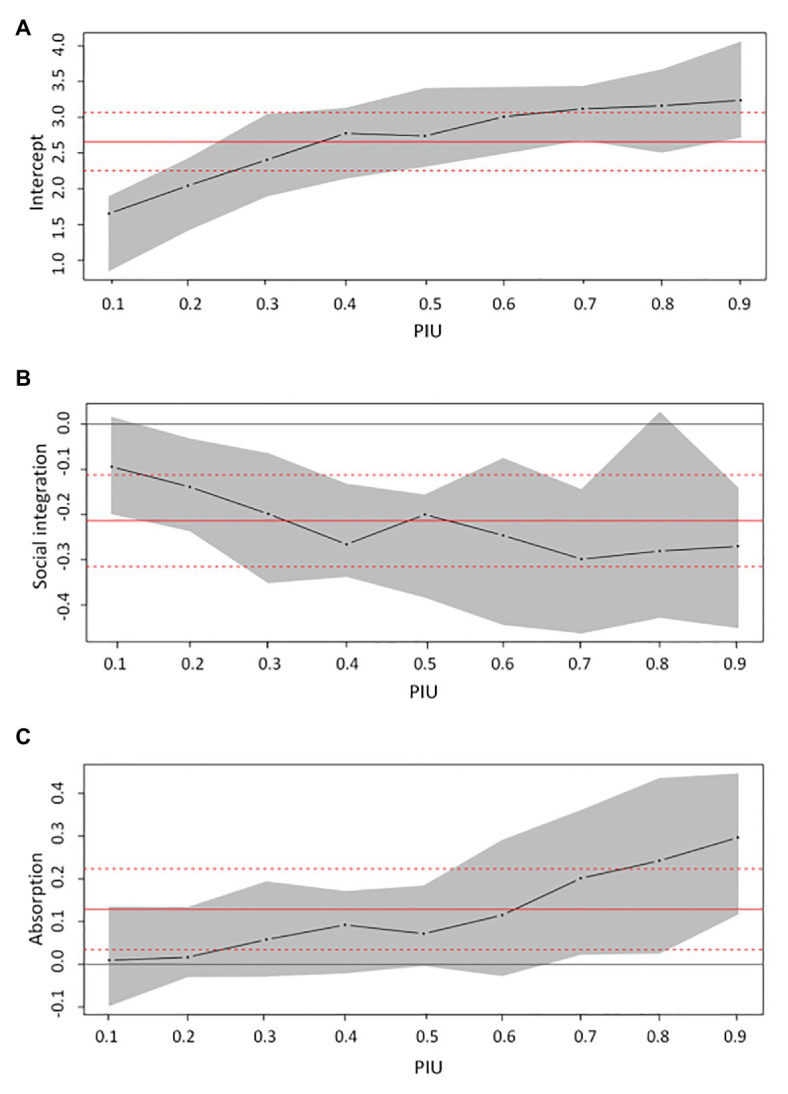
Quantile process plots for PIU on social integration and absorption. **(A)** intercept, **(B)** slope of social integration, and **(C)** slope of absorption. The dark line reflects the estimated coefficient conditional on the quantile of PIU. The red line reflects the coefficient by tradition linear regression.

Quantile regression by R software (Mostafaei et al., 2019) was further applied to demonstrate whether the level of PIU was related to the association between PIU and engaged living. The results of quantile regression are shown in Figures 1A–C. Social integration and absorption were strongly related to PIU when PIU increased from the 0.10 quantile to the 0.90 quantile. Specifically, for those adolescents in the *High-PIU* class (0.88 quantile for averaged PIU score, and 0.86~0.89 quantile for 95% CI), PIU was related to social integration and absorption. For the *Medium-PIU* class (0.45 quantile for *μ*, 0.4511~0.4518 for 95% CI), PIU was only associated with social integration. There was no association of PIU with social integration and absorption for adolescents in the *Low-PIU* class (0.11 quantile for *μ*, 0.106~0.108 quantile for 95% CI).

### Characteristics of Latent Classes

To examine the different demographics and psychological needs among the adolescents of each class, individuals were assigned to classes based on their most likely latent class membership and this membership was then treated as a known value to examine the characteristics of each latent class. As shown in Table 4, the three latent classes had no difference in age but there was a difference in sex; the proportion of males was higher among the *High-PIU* class (46.40%) with significant association of both social integration and absorption with PIU, followed by the *Medium-PIU* class (36.09%) with an association with social integration, and lastly by the *Low-PIU* class (24.48%), which had no association with engaged living. Additionally, the class-specific mean score for competence and autonomy were significantly higher for the *Low-PIU* class (*μ*s = 4.33, 4.52) than the *High-PIU* class (*μ*s = 4.09, 4.12) and the *Medium-PIU* class (*μ*s = 4.13, 4.29).

**Table 4:** Characteristics associated with latent classes.

Measure	Class 1: *High-PIU*	Class 2: *Medium-PIU*	Class 3: *Low-PIU*	*F* or *x*^2^	*Post hoc*	*M*	*SD*	*η*^2^
Age	15.74	15.60	15.49	0.78	—	2.94	1.82	0.003
Sex(Male)	46.40%	36.09%	24.48%	17.98^**^	C1 > C2 > C3	37.04%	—	—
Competence	4.09	4.13	4.33	4.62^**^	C1, C2 < C3	4.62	0.80	0.01
Autonomy	4.12	4.29	4.52	9.57^***^	C1, C2 < C3	4.28	0.86	0.29
Relatedness	5.23	5.30	5.41	1.76	—	5.30	0.88	0.01

^*^p < 0.05, ^**^p < 0.01, ^***^p < 0.001. Class 1, with the severest level of PIU, was defined by a negative association of social integration and a positive association of absorption with PIU. Class 2, with a medium level of PIU, was defined by a negative association of social integration and no significant association of absorption with PIU. Class 3, with the lowest level of PIU, was defined by no association of social integration or absorption with PIU.

## Discussion

By using a mixture regression model, this study identified three subgroups with different links between engaged living and PIU, which provides better understanding of the factors on PIU and could be used to create further guidance for effective interventions targeted to the particular class. Specifically, for adolescents with the highest level of PIU, increased PIU was associated with lower social integration and higher absorption; for adolescents with a medium level of PIU, increased PIU was only associated with lower social integration. No association has been found between PIU and engaged living for adolescents with the lowest PIU. For the classes with high and medium levels of PIU, their psychological needs for competence and autonomy had been less satisfied. Additionally, the proportion of males was highest in the *High-PIU* class, followed by the *Medium-PIU* class, and lastly by the *Low-PIU* class.

Compared with the standard linear regression model, mixture regression model detects the heterogeneous patterns of links between adolescents’ engaged living and PIU, which augmented the explained variance in the relationship between engaged living and PIU among adolescents. Notably, we found the different links were especially associated with the level of PIU. For adolescents with high or medium levels of PIU, low social integration was an associated factor for PIU, which further indicated that social integration can play an important role in prevention for adolescents with a relatively higher risk of PIU. This is consistent with previous findings that higher social skills and higher levels of social support are associated with lower PIU (Bolat et al., 2018; Chi-Ying, 2018; Prievara et al., 2018). Namely, being passionate about helping others, as the main characteristic of social integration, will expand and strengthen individuals’ social networks, and will provide more social support and sense of belonging in the real world (Davids et al., 2016), and thus make it easier to avoid internet addiction (Mo et al., 2018).

Among adolescents with a high level of PIU, high absorption was also associated with increased PIU. Although inconsistent with the previous assumption that absorption is related to positive outcomes, such as positive emotion (Froh et al., 2010), this is consistent with the findings supporting flow experience as a risk factor of PIU (Stavropoulos et al., 2018; Barnes et al., 2019). Flow represents the tendency to become totally absorbed in the activity at hand, which may produce positive emotions regardless of whether these activities are in the real world or not. Absorption also reflects autonomy when the goal is chosen personally, and is realizable either in the real world or the virtual one. The subjective gratification gained from an artificial world would drive an individual’s addiction to the internet (Stavropoulos et al., 2018). In line with a previous longitudinal study (Stavropoulos et al., 2018), we did not find that the association between PIU and absorption varies as a function of age for adolescents, which may indicate intervention targeting absorption to the internet would be generalized among adolescents of different ages.

In addition, the two classes with high and moderate levels of PIU had lower competence and autonomy, compared with those scoring low in PIU. This is similar to previous findings that internet addiction is associated with fewer psychological needs being satisfied (Liu et al., 2019). When psychological needs cannot be satisfied, self-motivation will be diminished, or be compensated for by shifting to another context (Liu et al., 2019; Scerri et al., 2019). The internet can be a very appealing and easily accessible way for young people to shift to another living context for the sake of satisfying their needs. For instance, they could promote themselves on social networking sites when they feel they are not good enough in real life (Islam et al., 2018). When self-motivation is driven by externalized factors (e.g., parents’ demand) rather than self-awareness, young people end up with lower sustained behavioral changes and less self-regulation of addictive behaviors (Kushnir et al., 2015). Moreover, we found a higher proportion of males for subgroups with more severe PIU, which is consistent with previous literature finding males with more severe PIU (Chi-Ying, 2018). Probably due to less developed social skills and a lower ability to cope with stress through communication, male adolescents are more likely to get addicted to online socialization or games (Lei et al., 2018).

Practically, the heterogeneous subgroups as identified in the current study indicated that intervention programs for PIU should be tailored to different subgroups. For adolescents with moderate or high levels of PIU, programs should be tailored based on their interests, to encourage them to engage in more social interaction offline (e.g., playing tennis in a tennis court with friends instead of playing tennis games online). Additionally, for subgroups with more psychological needs still to be satisfied, engaging in some public service activities would make them feel more confident and autonomous in problem solving, which would satisfy their need for competence and autonomy to some degree (Cui and Ji, 2019). For those with more severe PIU, with absorption as another potential risk factor, psychoeducation should be provided for them, to enable better understanding and increased self-awareness of online flow, in order to protect from absorption in the flow produced by the virtual world and the resultant disengagement from real life (Stavropoulos et al., 2018). Notably, male adolescents have higher proportions in the more severe subgroups, and therefore programs should adjust the emphasis according to boys’ interests and characteristics.

Nevertheless, a few limitations should be addressed in future studies. First, the current data were drawn from a cross-sectional study, and the predictive relationship between engaged living and PIU, as well as the potential moderating effect, should be further examined by longitudinal design. Second, future studies should further differentiate the definition of absorption between real and virtual life, so as to better examine their divergent associations with PIU. Third, considering the feasibility of assessment, the participants filled in the questionnaire with pencil and paper, without recording the exact duration of the survey. Some minor errors would be unavoidable, even though we double checked all the data input. Fourth, although with good or fair reliability for most of the measures, interpretation of the results should be taken cautiously, due to the poor validity of the measures in the current data, especially for psychological needs. In addition, although the construct of PIU in the current study has replicated that of the abbreviated version commonly used in other countries (Laconi et al., 2019), caution should also be paid when generalizing the conclusion to other populations.

Limitations notwithstanding, the current study contributes to the literature in identifying subgroups of adolescents with different levels of PIU, as well as its different associations with engaged living. The current findings suggest that prevention and intervention programs against PIU should be developed based on the different levels of PIU. The programs could also prioritize male adolescents of different ages, and focus on satisfying their psychological needs for competence and autonomy.

## Data Availability Statement

All datasets generated for this study are included in the article/supplementary material.

## Ethics Statement

The studies involving human participants were reviewed and approved by Four junior-senior high schools of Guangzhou. Written informed consent to participate in this study was provided by the participants’ legal guardian/next of kin.

## Author Contributions

JZ planned the study and wrote the manuscript. CJ conducted the data analysis. CY helped to plan the study, interpret the result with theory, and revise the manuscript. TQ made contribution to the literature review and summarization. ZL have made contributions to the revision (including data analysis and elaboration of the new results). All authors contributed to the article and approved the submitted version.

### Conflict of Interest

The authors declare that the research was conducted in the absence of any commercial or financial relationships that could be construed as a potential conflict of interest.

## References

[ref1] Ataşalar J. Michou A. (2019). Coping and mindfulness: mediators between need satisfaction and generalized problematic internet use. J. Media Psychol. Theories Methods App. 31, 110–115. 10.1027/1864-1105/a000230

[ref2] Baloğlu M. Kozan H. İ. Ö. Kesici Ş. (2018). Gender differences in and the relationships between social anxiety and problematic internet use: canonical analysis. J. Med. Internet Res. 20:e33. 10.2196/jmir.8947, PMID: 29367182 PMC5803528

[ref3] Barnes S. J. Pressey A. D. Scornavacca E. (2019). Mobile ubiquity: understanding the relationship between cognitive absorption, smartphone addiction and social network services. Comput. Hum. Behav. 90, 246–258. 10.1016/j.chb.2018.09.013

[ref4] Beard K. W. Wolf E. M. (2001). Modification in the proposed diagnostic criteria for internet addiction. Cyberpsychol. Behav. 4, 377–383. 10.1089/109493101300210286, PMID: 11710263

[ref5] Bolat N. Yavuz M. Kayı E. Zorlu A. (2018). The relationships between problematic internet use, alexithymia levels and attachment characteristics in a sample of adolescents in a high school, Turkey. Psychol. Health Med. 23, 604–611. 10.1080/13548506.2017.1394474, PMID: 29067840

[ref6] Chi-Ying C. (2018). Smartphone addiction: psychological and social factors predict the use and abuse of a social mobile application. Inf. Commun. Soc. 23, 454–467. 10.1080/1369118X.2018.1518469

[ref7] Cui D. Ji Q. (2019). What makes social Q&A site use enjoyable? The role of using modes and intrinsic needs satisfaction. Psychol. Pop. Media Cult. 8, 190–197. 10.1037/ppm0000177

[ref8] Davids E. L. Roman N. V. Kerchhoff L. J. (2016). Adolescent goals and aspirations in search of psychological well-being: from the perspective of self-determination theory. S. Afr. J. Psychol. 47, 121–132. 10.1177/0081246316653744

[ref9] Deci E. L. Ryan R. M. (2000). The “what” and “why” of goal pursuits: human needs and the self-determination of behavior. Psychol. Inq. 11, 227–268. 10.1207/S15327965PLI1104_01

[ref10] Fornell C. Larcker D. F. (1981). Structural equation models with unobservable variables and measurement error: algebra and statistics. J. Mark. Res. 18, 382–388. 10.1177/002224378101800313

[ref11] Froh J. J. Kashdan T. B. Yurkewicz C. Fan J. Allen J. Glowacki J. (2010). The benefits of passion and absorption in activities: engaged living in adolescents and its role in psychological well-being. J. Posit. Psychol. 5, 311–332. 10.1080/17439760.2010.498624

[ref12] Guo L. Luo M. Wang W. Huang G. Xu Y. Gao X.. (2018). Association between problematic internet use, sleep disturbance, and suicidal behavior in Chinese adolescents. J. Behav. Addict. 7, 965–975. 10.1556/2006.7.2018.115, PMID: 30474380 PMC6376369

[ref13] Hernández C. Ottenberger D. R. Moessner M. Crosby R. D. Ditzen B. (2019). Depressed and swiping my problems for later: the moderation effect between procrastination and depressive symptomatology on internet addiction. Comput. Hum. Behav. 37, 159–172. 10.1016/j.chb.2019.02.027

[ref14] Hu E. Stavropoulos V. Anderson A. Scerri M. Collard J. (2019). Internet gaming disorder: feeling the flow of social games. Addict. Behav. Rep. 9:100140. 10.1016/j.abrep.2018.10.004, PMID: 31193693 PMC6541905

[ref15] Islam A. K. M. N. Mäntymäki M. Benbasat I. (2018). Duality of self-promotion on social networking sites. Inf. Technol. People 32, 269–296. 10.1108/itp-07-2017-0213

[ref16] Kelley K. J. Gruber E. M. (2010). Psychometric properties of the problematic internet use questionnaire. Comput. Hum. Behav. 26, 1838–1845. 10.1016/j.chb.2010.07.018

[ref17] Kushnir V. Godinho A. Hodgins D. C. Hendershot C. S. Cunningham J. A. (2015). Motivation to quit or reduce gambling: associations between self-determination theory and the transtheoretical model of change. J. Addict. Dis. 35, 58–65. 10.1080/10550887.2016.1107315, PMID: 26488909

[ref18] Laconi S. Urbán R. Kaliszewska-Czeremska K. Kuss D. J. Gnisci A. Sergi I.. (2019). Psychometric evaluation of the nine-item problematic internet use questionnaire (PIUQ-9) in nine European samples of internet users. Front. Psych. 10:136. 10.3389/fpsyt.2019.00136, PMID: 30984037 PMC6448041

[ref19] Lanza S. T. Cooper B. R. Bray B. C. (2014). Population heterogeneity in the salience of multiple risk factors for adolescent delinquency. J. Adolesc. Health 54, 319–325. 10.1016/j.jadohealth.2013.09.007, PMID: 24231260 PMC3943167

[ref20] Lei H. Li S. Chiu M. M. Lu M. (2018). Social support and internet addiction among mainland Chinese teenagers and young adults: a meta-analysis. Comput. Hum. Behav. 85, 200–209. 10.1016/j.chb.2018.03.041

[ref21] Leménager T. Hoffmann S. Dieter J. Reinhard I. Mann K. Kiefer F. (2018). The links between healthy, problematic, and addicted internet use regarding comorbidities and self-concept-related characteristics. J. Behav. Addict. 7, 31–43. 10.1556/2006.7.2018.13, PMID: 29444606 PMC6035020

[ref22] Li D. Li X. Wang Y. Zhao L. Bao Z. Wen F. (2013). School connectedness and problematic internet use in adolescents: a moderated mediation model of deviant peer affiliation and self-control. J. Abnorm. Child Psychol. 41, 1231–1242. 10.1007/s10802-013-9761-9 23695186

[ref23] Li D. Zhang W. Li X. Zhen S. Wang Y. (2010). Stressful life events and problematic internet use by adolescent females and males: a mediated moderation model. Comput. Hum. Behav. 26, 1199–1207. 10.1016/j.chb.2010.03.031

[ref24] Liu J. S. Lin L. L. Lu Y. Wei C. B. Zhou Y. Chen X. Y. (2013). Reliability and validity of the Chinese version of the basic psychological needs scale. Chin. J. Mental Health 27, 791–795.

[ref25] Liu Q. Lin Y. Zhou Z. Zhang W. (2019). Perceived parent–adolescent communication and pathological internet use among Chinese adolescents: a moderated mediation model. J. Child Fam. Stud. 28, 1571–1580. 10.1007/s10826-019-01376-x

[ref26] Masten A. S. (2001). Ordinary magic: resilience processes in development. Am. Psychol. 56, 227–238. 10.1037/0003-066x.56.3.227 11315249

[ref27] Mo P. K. H. Chan V. W. Y. Chan S. W. Lau J. T. F. (2018). The role of social support on emotion dysregulation and internet addiction among Chinese adolescents: a structural equation model. Addict. Behav. 82, 86–93. 10.1016/j.addbeh.2018.01.027 29501012

[ref28] Mostafaei S. Kabir K. Kazemnejad A. Feizi A. Mansourian M. Hassanzadeh Keshteli A.. (2019). Explanation of somatic symptoms by mental health and personality traits: application of Bayesian regularized quantile regression in a large population study. BMC Psychiatry 19:207. 10.1186/s12888-019-2189-1, PMID: 31269925 PMC6610832

[ref29] Ni X. Qian Y. Wang Y. (2017). Factors affecting pathological internet use among Chinese university students. Soc. Behav. Personal. Int. J. 45, 1057–1068. 10.2224/sbp.6039

[ref30] Park S. Kang M. Kim E. (2014). Social relationship on problematic internet use (PIU) among adolescents in South Korea: a moderated mediation model of self-esteem and self-control. Comput. Hum. Behav. 38, 349–357. 10.1016/j.chb.2014.06.005

[ref31] Philippe F. L. Vallerand R. J. Lavigne G. L. (2009). Passion does make a difference in people’ s lives: a look at well-being in passionate and non-passionate individuals. Health 1, 3–22. 10.1111/j.1758-0854.2008.01003.x

[ref32] Prievara D. K. Piko B. F. Luszczynska A. (2018). Problematic internet use, social needs, and social support among youth. Int. J. Ment. Heal. Addict. 17, 1008–1019. 10.1007/s11469-018-9973-x

[ref33] Rivkin W. Diestel S. Schmidt K. (2018). Which daily experiences can foster well-being at work? A diary study on the interplay between flow experiences, affective commitment, and self-control demands. J. Occup. Health Psychol. 23, 99–111. 10.1037/ocp0000039 27101337

[ref34] Scerri M. Anderson A. Stavropoulos V. Hu E. (2019). Need fulfilment and internet gaming disorder: a preliminary integrative model. Addict. Behav. Rep. 9:7. 10.1016/j.abrep.2018.100144, PMID: 31193898 PMC6543453

[ref35] Séguin-Lévesque C. Lyne M. Lalibertea N. Pelletier L. G. Blanchard C. Vallerand R. J. (2003). Harmonious and obsessive passion for the internet: their associations with the couple’s relationship. J. Appl. Soc. Psychol. 33, 197–221. 10.1111/j.1559-1816.2003.tb02079.x

[ref36] Sha P. Sariyska R. Riedl R. Lachmann B. Montag C. (2019). Linking internet communication and smartphone use disorder by taking a closer look at the facebook and whatsapp applications. Addict. Behav. Rep. 9:9. 10.1016/j.abrep.2018.100148 PMC6543448 31193857

[ref37] Stavropoulos V. Griffiths M. D. Burleigh T. L. Kuss D. J. Doh Y. Y. Gomez R. (2018). Flow on the internet: a longitudinal study of internet addiction symptoms during adolescence. Behav. Inform. Technol. 37, 159–172. 10.1080/0144929x.2018.1424937

[ref38] Tateno M. Teo A. R. Ukai W. Kanazawa J. Katsuki R. Kubo H.. (2019). Internet addiction, smartphone addiction, and Hikikomori trait in Japanese young adult: social isolation and social network. Front. Psych. 10:455. 10.3389/fpsyt.2019.00455, PMID: 31354537 PMC6635695

[ref39] Tian L. Han M. Huebner E. S. (2014). Preliminary development of the adolescent students’ basic psychological needs at school scale. J. Adolesc. 37, 257–267. 10.1016/j.adolescence.2014.01.005 24636686

[ref40] Young K. S. (1998). Internet addiction: the emergence of a new clinical disorder. Cyberpsychol. Behav. 1, 237–244. 10.1089/cpb.1998.1.237

[ref41] Zhang Y. Wang D. Gao X. Cai Y. Tu D. (2019). Development of a computerized adaptive testing for internet addiction. Front. Psychol. 10:1010. 10.3389/fpsyg.2019.01010, PMID: 31133939 PMC6514228

[ref42] Zhang J. Zhang M. Zhang W. Jiao C. (2014). Model selection for complex multilevel latent class model. Commun. Stat. Simul. Comput. 43, 838–850. 10.1080/03610918.2012.718836

[ref43] Zhou Y. Li D. Li X. Wang Y. Zhao L. (2017). Big five personality and adolescent internet addiction: the mediating role of coping style. Addict. Behav. 64, 42–48. 10.1016/j.addbeh.2016.08.009, PMID: 27543833

